# *Klebsiella pneumoniae* causes mammary gland damage via FNIP1-mediated mitochondrial dysfunction

**DOI:** 10.1093/jas/skaf384

**Published:** 2025-11-06

**Authors:** Pengfei Dong, Changning Yuan, Zhihao Wang, Peng Mao, Kangjun Liu, Jianji Li, Junsheng Dong, Luying Cui, Long Guo, Xia Meng, Guoqiang Zhu, Hongyun Liu, Ran Wang, Lili Zhang, Heng Wang

**Affiliations:** College of Veterinary Medicine, Yangzhou University, Jiangsu Co-innovation Center for Prevention and Control of Important Animal Infectious Diseases and Zoonoses, Yangzhou, China; College of Veterinary Medicine, Yangzhou University, Jiangsu Co-innovation Center for Prevention and Control of Important Animal Infectious Diseases and Zoonoses, Yangzhou, China; Pancreatic Center, Department of Gastroenterology, Yangzhou Key Laboratory of Pancreatic Disease, The Affiliated Hospital of Yangzhou University, Yangzhou University, Yangzhou, China; College of Veterinary Medicine, Yangzhou University, Jiangsu Co-innovation Center for Prevention and Control of Important Animal Infectious Diseases and Zoonoses, Yangzhou, China; College of Veterinary Medicine, Yangzhou University, Jiangsu Co-innovation Center for Prevention and Control of Important Animal Infectious Diseases and Zoonoses, Yangzhou, China; College of Veterinary Medicine, Yangzhou University, Jiangsu Co-innovation Center for Prevention and Control of Important Animal Infectious Diseases and Zoonoses, Yangzhou, China; College of Veterinary Medicine, Yangzhou University, Jiangsu Co-innovation Center for Prevention and Control of Important Animal Infectious Diseases and Zoonoses, Yangzhou, China; College of Veterinary Medicine, Yangzhou University, Jiangsu Co-innovation Center for Prevention and Control of Important Animal Infectious Diseases and Zoonoses, Yangzhou, China; College of Veterinary Medicine, Yangzhou University, Jiangsu Co-innovation Center for Prevention and Control of Important Animal Infectious Diseases and Zoonoses, Yangzhou, China; College of Veterinary Medicine, Yangzhou University, Jiangsu Co-innovation Center for Prevention and Control of Important Animal Infectious Diseases and Zoonoses, Yangzhou, China; College of Veterinary Medicine, Yangzhou University, Jiangsu Co-innovation Center for Prevention and Control of Important Animal Infectious Diseases and Zoonoses, Yangzhou, China; Institute of Dairy Science, Zhejiang University, Hangzhou, China; Institute of Food Safety and Nutrition, Jiangsu Academy of Agricultural Sciences, Nanjing, China; Institute of Food Safety and Nutrition, Jiangsu Academy of Agricultural Sciences, Nanjing, China; College of Veterinary Medicine, Yangzhou University, Jiangsu Co-innovation Center for Prevention and Control of Important Animal Infectious Diseases and Zoonoses, Yangzhou, China

**Keywords:** Bovine mastitis, folliculin interacting protein 1, *Klebsiella pneumoniae*, milk fat and protein synthesis, mitochondrial function

## Abstract

*Klebsiella pneumoniae* (*K. pneumoniae*) is one of the pathogens causing clinical mastitis of bovine. Previous studies have demonstrated that mitochondrial damage and dysfunction are important mechanisms of mastitis in dairy cattle. Folliculin interacting protein 1 (FNIP1) is a major metabolic regulator of mitochondrial function with proinflammatory capabilities, but its role in *K. pneumoniae*-induced mastitis is yet to be elucidated. Thus, the studies were conducted to clarify the role of FNIP1-mediated mitochondrial function in mastitis caused by *K. pneumoniae* in vivo and in vitro. The experiments verified that *K. pneumoniae* caused decrease of milk fat and protein synthesis evidently in the mammary glands and bovine mammary epithelial cells (BMECs), accompanied by an imbalance in mitochondrial fission and fusion, increased mitochondrial permeability transition pore opening, decreased membrane potential and ATP content. While the enhancement of mitochondrial function alleviated *K. pneumoniae*-induced BMECs injury via relieving milk fat and protein dyssynthesis. Notably, transcriptomic analysis revealed that FNIP1 expression was upregulated in BMECs induced by *K. pneumoniae*. Further investigations revealed FNIP1 silencing improved milk synthesis by alleviating mitochondrial dysfunction caused by *K. pneumoniae* infection, and further inhibiting the activation of inflammatory factors, which in turn prompted the mammary recovery. In conclusion, *K. pneumoniae* inhibited mitochondrial function by activating FNIP1, which reducing the synthesis of milk fat and protein, thereby in turn lowers milk quality and induced mastitis. This study showed that FNIP1 has the potential as a novel target for the prevention and control of bovine mastitis.

## Teaser

The milk synthesis was inhibited in the mastitis induced by *K. pneumoniae*, which depended on FNIP1-mediated mitochondrial dysfunction.

## Introduction

Bovine mastitis, as a substantial mammary disease, not only profoundly affects the health status of dairy cows but also leads to the global decline in raw milk quality and further poses a potential risk to public health safety (Klaas and Zadoks, 2018; Panchal et al., 2024). *Klebsiella pneumoniae* (*K. pneumoniae*), the Gram-negative bacteria, is an environmental pathogen that widely present in soil, feces and sewage (Kowalczyk et al., 2022). *K. pneumoniae* has been identified as an important bacterial trigger of the mastitis in dairy cows by damage to bovine mammary epithelial cells (BMECs), altering milk quality, and causing inflammatory changes in the udder (Cheng et al., 2020; Zheng et al., 2022). The important function of BMECs is the synthesis of milk fat and protein. It has been shown that lipopolysaccharide, a major endotoxin of Gram-negative bacteria, reduced casein in the milk and inhibited triglyceride synthesis in BMECs via the transcription factor sterol regulatory element-binding protein 1 (SREBP1), which led to the depression of milk fat synthesis (Wang et al., 2018b; Geng et al., 2021). However, the mechanism of *K. pneumoniae* inhibiting milk fat and protein synthesis in BMECs remains to be fully elucidated.

Mitochondria act as the principal energy-generating organelles in cells, governing homeostasis and lipid metabolism (Nunnari and Suomalainen, 2012; Benador et al., 2019), and their dysfunction causes bioenergy failure, oxidative stress, inflammation, and cell death (Monzel et al., 2023). The normal functioning of mitochondria depends not only on the oxidative phosphorylation complexes within the mitochondrial respiratory chain but also crucially on the two key processes of mitochondrial dynamics, mitochondrial fusion and fission. These two processes are primarily regulated by proteins such as optic atrophy 1 (OPA1), mitofusin 1 (MFN1), mitofusin 2 (MFN2), dynamin-related protein 1 (DRP1), and mitochondrial fission 1 (FIS1) (Chan, 2020; Chen et al., 2023; Quintana-Cabrera and Scorrano, 2023). A previous study proposed that heat stress disrupted the equilibrium between mitochondrial division and fusion, which in turn led to impaired mammary epithelial cells function in dairy cows (Chen et al., 2020). Mitochondrial homeostasis plays central roles in both reactive oxygen species disposal and ATP production, maintaining normal function of tissues and organs (Nolfi-Donegan et al., 2020; Chakrabarty and Chandel, 2021). Studies have shown that the imbalance of mitochondrial homeostasis lead to enhanced mammary oxidative stress and impaired milk yield (Cheng and Ristow, 2013; Favorit et al., 2021). Therefore, it is necessary to investigate whether mitochondria are involved in regulating mammary gland injury caused by *K. pneumoniae* and examined its molecular mechanisms and functions.

Folliculin interacting protein 1 (FNIP1) has recently been recognized as a pivotal regulator of cellular mitochondrial function (Malik et al., 2023; Xiao et al., 2024; Zeng et al., 2024; Ran et al., 2025). It plays a crucial role in modulating mitochondrial function and eliminating damaged mitochondria (Freemantle and Hardie, 2023; Malik et al., 2023). Xiao et al. showed that FNIP1 regulated muscle mitochondrial biogenesis and quality control programs via AMPK (Xiao et al., 2021). FNIP1 knockout in skeletal muscle resulted in increased mitochondrial content and augmented metabolic capacity (Xiao et al., 2024). Moreover, a study has demonstrated that loss of FNIP1 led to marked mitochondrial biogenesis and augmented mitochondrial respiration capacity (Yin et al., 2022). Increasing evidence have proved that FNIP1 is associated with a wide variety of cellular processes, such as mitochondrial quality control, alteration of mitochondrial morphology, reductive stress response, reduction oxidative phosphorylation, and protection cells from unwarranted ROS accumulation (Woodford et al., 2016; Manford et al., 2020; Manford et al., 2021). Our previous research has shown that *K. pneumoniae* infection causes inflammation and oxidative stress in BMECs (Ma et al., 2022). However, whether FNIP1 participates in the regulation of milk fat and protein synthesis during the infection induced by *K. pneumoniae* and what role FNIP1 plays in mitochondrial function have not been researched yet in the mastitis. This study focused on the mechanism of FNIP1 in the regulation of mitochondrial function, milk lipid and protein synthesis.

In this study, we established a model of the bovine mammary gland injury induced by *K. pneumoniae* to explore the role and associated mechanisms of FNIP1 in the mastitis. Here, the study reported that *K. pneumoniae* impaired mitochondrial function and inhibited milk fat and protein synthesis through activation of FNIP1 signaling, which led to inflammation and caused mastitis. The findings may provide new direction for the prevention of mastitis in dairy cows caused by bacterial infections.

## Materials and Methods

### The IACUC statement

The animal experimental protocol was approved by the Animal Care and Ethics Committee of the Yangzhou University (Approval ID: 202205130) and complied strictly with the ARRIVE guidelines (https://arriveguidelines.org/).

### Reagents

DMEM/F12 and L-glutamine were purchased from Sigma-Aldrich (St. Louis, MO, USA). Fetal bovine serum (FBS) was acquired through Gibco BRL Company (New York, USA). BCA protein assay kit (W9811) was obtained from TIANGEN (Beijing, China). BODIPY 493/503 staining kit and DAPI (P0131) were obtained from Beyotime (Shanghai, China). JC-1 apoptosis detection kit (KGA1904-100) was obtained from Keygen BIO (Jiangsu, China). Mitochondrial permeability transition pore assay (mPTP) (KTA4002) and CheKine Micro Na^+^/K^+^-ATPase activity assay kit (KTB1800) were purchased from Abbkine Scientific Co., Ltd (Hubei, China). MitotrackerTM red CMXRos (M7512) was provided by Thermo Fisher Scientific (CA, USA). The following primary antibodies were used: SREBP1 (14088-1-AP, Proteintech, Hubei, China), β-casein (HY-P81091, MCE, Shanghai, China), OPA1 (DF8587, Affinity, Jiangsu, China), MFN1 (13798-1-AP, Proteintech, Hubei, China), cytochrome c oxidase I (COX I) (DF8920, Affinity, Jiangsu, China), DRP1 (12957-1-AP, Proteintech, Hubei, China), FIS1 (10956-1-AP, Proteintech, Hubei, China), FNIP1 (28380-1-AP, Proteintech, Hubei, China), β-actin (AF7018, Affinity, Jiangsu, China).

### Preparation of *K. pneumoniae*

The *K. pneumoniae* originated from milk samples of cows with mastitis and was stored in our laboratory. The strain belongs to a hypermucoviscous (HMV) phenotype and has *rmpA*, *wabG*, *uge*, and *fimH* genes (Ma et al., 2022; Xu et al., 2024). These bacteria were cultured in LB medium at 37 °C and collected during their logarithmic growth phase. Subsequently, based on the multiplicity of infection (MOI = 10) required for the experiment, the bacteria were diluted to an appropriate concentration using cell culture medium.

### Cell culture

BMECs were cultured as described previously and cryopreserved in liquid nitrogen (Wang et al., 2018a). BMECs were cultured in DMEM/F12 medium containing 15% FBS and 1 mmol/L L-glutamine at 37 °C, 5% CO_2_.

### Cell treatment

At a cell density of 80%, cells were infected with *K. pneumoniae* at MOI of 10 for 0, 2, 4, and 6 h. For inhibition studies, BMECs underwent pretreatment with 10 μM Mdivi-1 (MedChem Express, USA) for 2 h, and then infected with *K. pneumoniae* at MOI of 10 for 6 h.

### Silencing of FNIP1

Transfection was performed using lentiviral vectors (LV) (Genemoditech, Shanghai, China) carrying shFNIP1 (sh-FNIP1) and a negative control (sh-NC) to achieve knockdown of FNIP1. The sequences of sh-FNIP1 and sh-NC are shown in Table 1. Firstly, BMECs were seeded into six-well plates and cultured until they reached approximately 50% confluence. Subsequently, according to the operation manual, the cells were transfected with either sh-FNIP1 or sh-NC. The silencing efficiency of FNIP1 was then evaluated by Western blotting analysis.

**Table 1.** The sequences of sh-NC and sh-FNIP1

Gene	TargetSeq
*sh-NC*	TTCTCCGAACGTGTCACGT
*sh-FNIP1-1*	TCAGGACAACAGTACATTAAA
*sh-FNIP1-2*	CTCTCAGCAGCTTGCTTATTA
*sh-FNIP1-3*	GAAAGCCACATGAACAAATTA

### Animal experiment

The experimental protocol was approved by the Animal Care and Ethics Committee of Yangzhou University (Approval ID: 202205130). The experiment was conducted at experimental cattle ranch of Yangzhou University in Yangzhou. Six Holstein cows (approximately 270 d postpartum, milk yield 23.1 ± 2.8 kg/d, parity 2.5 times) were used in this study. Cows were screened according to the following criteria. All experimental cows were healthy with normal lactation function, no antibiotic treatment history in the month before the experiment, and the somatic cell count in milk before the experiment under 2 × 10^5^/mL for 5 consecutive days. After teats were disinfected, 5 mL sterile saline containing 10^8^ CFU/mL *K. pneumoniae* were introduced into the left rear quarter (*n* = 6) as the infection group, and another 5 mL sterile saline into the right rear quarter (*n* = 6) as the control group. After 24 h post infection, cows were anesthetized and mammary glands were collected surgically. Part of the mammary glands were fixed in 4% formaldehyde solution, and the rest of the tissues were stored at −80 °C after packaging for the following experiments.

### Quantitative real-time PCR

Total RNA from mammary gland tissues and BMECs was extracted by Trizol method, reverse transcription was performed using the RT reagents (R433, Vazyme, CN). SYBR Green fluorescent dye (Q712, Vazyme, CN) was used, the reaction program was set up, and finally the data were analyzed. Primer data are shown in Table 2.

**Table 2.** Primer sequences with their corresponding PCR product size

Gene	Primer (5′-3′)	Product (bp)
*β-actin*	CATCACCATCGGCAATGAGC	156
	AGCACCGTGTTGGCGTAGAG	
*TNF-α*	GGGCTTTACCTCATCTACTCACAG	132
	GATGGCAGACAGGATGTTGACC	
*IL-1β*	AGGTCCATACCTGACGGCTA	134
	TTGGGTGTCTCAGGCATCTC	
*IL-6*	TGAAAGCAGCAAGGAGACACT	90
	TGATTGAACCCAGATTGGAAGC	
*SREBP1*	CTCCGACACCACCAGCATCAAC	122
	GCAGCCCATTCATCAGCCAGAC	
*β-casein*	ACAGCAGCAAACAGAGGATGAACTC	148
	AGGCGGCACCACCACAGG	
*FNIP1*	TTATGGAGGAGAGCAGGAGGATTGG	132
	GAGCAATAGCCACCCAGCAAGG	

### Western blotting analysis

Total proteins were extracted from mammary gland tissues and BMECs, and their concentrations were determined by the BCA method. An equal amount of total protein was taken and separated by SDS-PAGE gel. Proteins were transferred to PVDF membrane. The membrane was blocked with 5% BSA for 1 h and then incubated with primary antibody at 4 °C overnight. The secondary antibody was incubated for 1 h. Finally, the signal was detected by Chemidoc XRS (Bio-Rad, Marnes-La Coquette, France).

### Histopathological observation and immunohistochemical analysis

Fixed mammary glands were embedded in paraffin and sections were prepared, which were stained with hematoxylin-eosin (H&E) and observed by light microscope. Mammary pathological changes were assessed using a semi-quantitative scale ranging from 0 to 11, and the scoring criteria were shown in Table 3. For each section, three randomly selected fields were observed, and the average score was calculated. Mammary sections were used for exploring the expression of FNIP1 protein and the typical immunohistochemical labeling process was performed.

**Table 3.** Inflammatory score of mammary gland tissues

Feature	Description	Score
Hyperemia/Edema	Normal	0
	Mild	1
	Moderate	2
	severe	3
Milk stasis/Acinar necrosis	Normal	0
	Milk	1
	Moderate	2
	Severe	3
Infiltration with neutrophil	0–1 Acinar or mammary gland neutrophil	0
	2–5 Acinar or mammary gland neutrophil	1
	6–10 Acinar or mammary gland neutrophil	2
	11–15 Acinar or mammary gland neutrophil	3
	16–20 Acinar or mammary gland neutrophil	4
	> 20 Acinar or mammary gland neutrophil	5

### Lipid droplets detection with BODIPY staining

Intracellular lipid droplets were stained with BODIPY 493/503 dye and incubated for 30 min. Subsequently, excess BODIPY dye was washed away with PBS, and the samples were then fixed with 4% PFA at 4 °C for 8 min. Image acquisition was performed on a Leica confocal laser scanning microscope (TCS SP2, Germany).

### Transcriptome sequencing

Total RNA from BMECs was extracted using TRIZOL reagent (R701, Vazyme, CN). After identification by Agilent 2100 bioanalyzer, the identified RNA was used to construct an RNA library. Transcriptome sequencing of the constructed RNA libraries was conducted on the Illumina sequencing platform. HISAT2 software mapped the readings to the bovine genome (NCBI: GCF_000298355.1). Gene expression levels were quantified employing the feature Counts tool from the subread package in conjunction with string tie. EdgeR and DESeq2 R packages were used for differential expression analysis. False discovery rate (FDR) < 0.05 and |log2 (fold change)| > 1.5 were considered significant. The results were visualized using the “pheatmap” and “ggplot2” R packages. In DAVID database (david.ncifcrf.gov/, 25 December 2024, accessed), Kyoto Encyclopedia Gene and Genome (KEGG) enrichment analysis of differentially expressed genes (DEGs) were performed, and *p *< 0.05 was used as the screening condition to screen the results with statistical differences. Result of KEGG enrichment analysis were visualized using the “ggplot2” R package.

### Mitochondrial function analysis

After undergoing the corresponding treatments, BMECs were incubated with JC-1 staining solution for 30 min, followed by washing BMECs. Subsequently, the red and green fluorescence intensities generated by JC-1 were detected and recorded using a flow cytometer. Finally, FlowJo software was used to analyze the changes of mitochondrial membrane potential (ΔΨm).

We evaluated the opening degree of mPTP in BMECs using an mPTP detection kit. BMECs were first incubated with calcein AM, followed by the addition of cobalt chloride to quench the fluorescence. Flow cytometry was then used for monitoring, and the obtained data were analyzed using FlowJo software.

ATP concentrations in the cow mammary tissues and BMECs were assessed using CheKine Micro Na^+^/K^+^-ATPase activity assay kit. The measurement of ATP levels was conducted using the Luminoskan Ascent luminometer from Thermo Fisher Scientific Inc., USA.

BMECs were cultured in 35-mm confocal dishes and infected with *K. pneumoniae*. To label mitochondria, BMECs were cultured with MitoTracker (200 nM) for 30 min at 37 °C. Confocal microscope (Leica TCS SPE, Germany) was used to obtain images.

After infected with *K. pneumoniae* for 6 h, BMECs were collected and made fixation with 2.5% glutaraldehyde, stained with osmium tetroxide, captured in 4% agarose, dehydrated with ethanol and wrapped in spurr-resin. Finally, double staining on 60 nm ultrathin sections was conducted with uranyl acetate and lead citrate. The cells were observed by transmission electron microscope (TEM).

### Statistical analysis

Data in this study were stated as mean ± SEM. Comparisons among different treatments were evaluated by *t* test and one-way analysis of variance (ANOVA). Graphs were generated using GraphPad Prism 9. *P *< 0.05 showed statistical significance.

## Results

### *K. pneumoniae* inhibited milk fat and protein synthesis of BMECs

**Figure 1.**
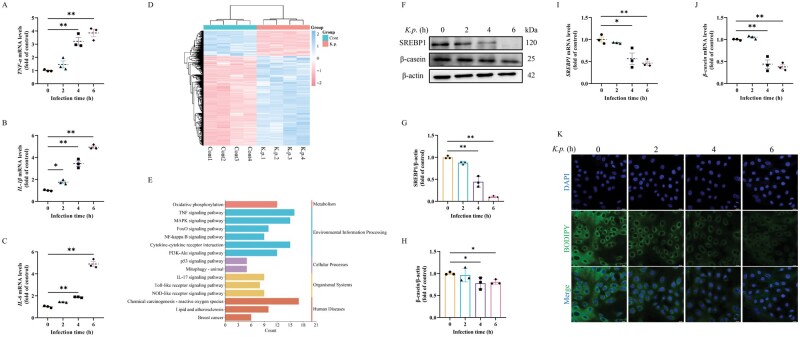
*K. pneumoniae* inhibited milk fat and protein synthesis in BMECs. (A–C) mRNA levels of *TNF-α*, *IL-1β*, and *IL-6* were detected by RT-qPCR method in BMECs (mean ± SEM, *n* = 3). (D) DEGs heat maps. (E) Enrichment analysis of differentially expressed genes KEGG. (F) The protein levels of *SREBP1* and *β-casein* were detected in BMECs. (G, H) Relative protein abundance of *SREBP1* and *β-casein* were normalized to β-actin (mean ± SEM, *n* = 3). (I, J) mRNA levels of *SREBP1* and *β-casein* were detected by RT-qPCR method in BMECs (mean ± SEM, *n* = 3). (K) Typical images of BODIPY 493/503 staining in BMECs. *P < 0.05; **P < 0.01.

To investigate the potential mechanism of *K. pneumoniae* infection-induced damage to BMECs, BMECs were stimulated with *K. pneumoniae* at MOI of 10 for 2, 4, and 6 h, and examined the expression of relevant inflammatory genes. The results showed that at these three time points, the mRNA levels of *TNF-α*, *IL-1β*, and *IL-6* genes all increased (Figure 1A–C). Therefore, transcriptomic analysis was performed at 6 h after *K. pneumoniae* infection in BMECs. Heat map result showed that 1878 DEGs were identified with fold-change ≥ 1.5 (*P *< 0.05) at 6 h infection with *K. pneumoniae*, which exhibited by 1464 upregulated and 414 downregulated DEGs (Figure 1D). Importantly, the KEGG analysis of DEGs indicated that genes enriched in mitophagy, lipid and oxidative phosphorylation were abnormally expressed in *K. pneumoniae*-infected BMECs (Figure 1E). Pathway analysis of DEGs was performed based on the KEGG database, and the enrichment results indicated that biological processes involving mitochondrial function and lipid metabolism are essential in the process of *K. pneumoniae* damage to BMECs.

SREBP1 functions as a canonical transcriptional master regulator orchestrating lipogenesis. As shown in Figure 1F, the expression of SREBP1 protein in BMECs was significantly decreased by *K. pneumoniae* infection at 2, 4, and 6 h (Figure 1F and G). Next, in studying the impact of *K. pneumoniae* on milk protein synthesis, we found that the protein level of β-casein decreased after BMECs were infected with *K. pneumoniae* (Figure 1F and H). After *K. pneumoniae* invasion, the mRNA levels of *SREBP1* and *β-casein* were distinctly decreased (Figure 1I and J). In addition, immunostaining images showed that fewer lipid droplets were detected in the *K. pneumoniae* infected group compared to the control group (Figure 1K). The above results confirmed that *K. pneumoniae* inhibited BMECs milk fat synthesis and milk protein synthesis.

### *K. pneumoniae* induced mitochondrial damage in BMECs

**Figure 2.**
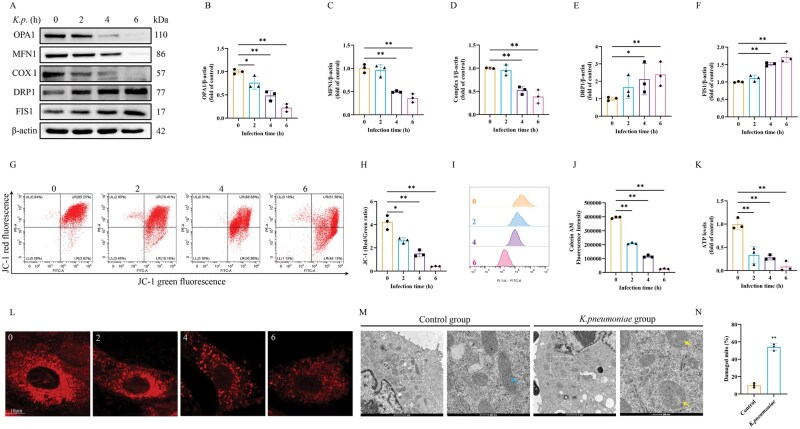
*K. pneumoniae* induced mitochondrial dynamic imbalance and mitochondrial quality control failure in BMECs. (A) The protein levels of OPA1, MFN1, COX I, DRP1, and FIS1 were detected in BMECs. (B–F) Relative protein abundance of OPA1, MFN1, COX I, DRP1, and FIS1 were normalized to β-actin (mean ± SEM, *n* = 3). (G) Flow cytometry was used to measure mitochondrial membrane potential level. (H) Changes in mitochondrial membrane potential were determined by the JC-1 red to green fluorescence ratio (mean ± SEM, *n* = 3). (I) The mPTP opening status was evaluated in BMECs by calcein-AM fluorescence. Representative flow cytometry graph and (J) statistical analysis of calcein-AM fluorescence intensity (mean ± SEM, *n* = 3). (K) Relative ATP levels (mean ± SEM, *n* = 3). (L) Mitochondrial morphology of BMECs. The mitochondria were labeled with MitoTracker. (M) Representative transmission electron microscope images of mitochondria. Blue arrow indicates normal mitochondria. Yellow arrow indicates defects in the mitochondrial cristae structure. (N) The percentage of damaged mitochondria was quantified, at least 100 mitochondria. *P < 0.05; **P < 0.01.

Mitochondria are key organelles involved in energy synthesis, and transcriptome analysis has been enriched to mitochondrial functional pathways. Therefore, the effect of *K. pneumoniae* on the protein levels of OPA1, MFN1, COX I, DRP1, and FIS1 were examined, which are closely related to mitochondrial function. The results showed that the protein levels of OPA1, MFN1, and COX I were significantly downregulated in the *K. pneumoniae* infection group, compared with the control group. In contrast, the protein levels of DRP1 and FIS1 were significantly upregulated in BMECs infected with *K. pneumoniae* (Figure 2A–F). Then, the key indices of mitochondrial function were also examined. JC-1 staining assay showed a decrease in the red/green ratio after *K. pneumoniae* infection of BMECs, indicating a decrease in ΔΨm (Figure 2G and H). Mitochondrial dysfunction is commonly associated with the opening of the mPTP. The results showed that exposure to *K. pneumoniae* results in decreased mitochondrial calcineurin fluorescence, indicating the opening of the mPTP (Figure 2I and J). Mitochondrial dynamics are closely related to energy metabolism, and a decrease in ATP concentration affects mitochondrial morphology. Measurements of ATP concentration in BMECs revealed a significant reduction in ATP levels following *K. pneumoniae* infection (Figure 2K). Moreover, infection with *K. pneumoniae* also led to disruption and fragmentation of mitochondria (Figure 2L). To assess the effect of *K. pneumoniae* infection on mitochondria in more depth, TEM was used to examine mitochondrial morphology. TEM images revealed that mitochondrial swelling and cristae degeneration in *K. pneumoniae*-infected BMECs were markedly more severe than those in the control group (Figure 2M and N). These results suggested that *K. pneumoniae* caused mitochondrial damage in BMECs. Given these results, mitochondrial dysfunction may play a role in *K. pneumoniae*-induced dyssynthesis of milk fat and protein, as well as in inflammatory responses.

### *K. pneumoniae* caused mitochondrial dynamic imbalance and milk dyssynthesis in mammary glands

**Figure 3.**
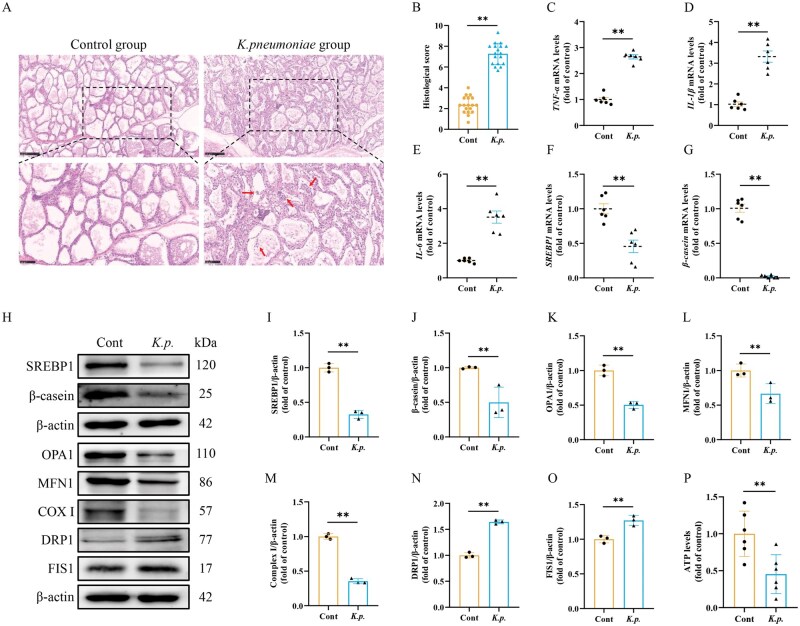
*K. pneumoniae* caused mitochondrial dysfunction in mammary gland and dyssynthesis of milk fat and protein. (A) Representative images of H&E staining. (B) The severity of mastitis was assessed by differences in histological score (*n* = 6 cows per group) between the control and *K. pneumoniae* infection groups. (C–G) mRNA levels of *TNF-α*, *IL-1β*, *IL-6, SREBP1*, and *β-casein* were detected by RT-qPCR method in mammary gland tissues (mean ± SEM, *n* = 6). (H) The protein levels of SREBP1, β-casein OPA1, MFN1, COX I, DRP1, and FIS1 were detected in mammary gland tissues. (I–O) Relative protein abundance of OPA1, MFN1, COX I, DRP1, FIS1, SREBP1, and β-casein were normalized to β-actin (mean ± SEM, *n* = 3). (P) Relative ATP levels (mean ± SEM, *n* = 6). *P < 0.05; **P < 0.01.

To further verify the above results, we conducted the experiment in vivo. Histopathological findings revealed that inflammatory cells infiltrated and mammary epithelial cells exfoliated in mammary glands infected with *K. pneumoniae* (Figure 3A). Histological score results showed that the score in *K. pneumoniae* infection group was significantly higher than that in the control group (Figure 3B). We also evaluated the effect of *K. pneumoniae* on inflammatory factors in mammary glands. The results showed that the mRNA levels of *TNF-α*, *IL-1β*, and *IL-6* in mammary glands invaded by *K. pneumoniae* were significantly elevated (Figure 3C–E). Consistent with the results *in vitro*, *SREBP1* and *β-casein* mRNA levels were also significantly decreased (Figure 3F and G). Western blot results further displayed that the protein levels of SREBP1 and β-casein were significantly inhibited (Figure 3H–J). These results indicated that obstruction of milk fat and protein synthesis were proved in bovine mastitis induced by *K. pneumoniae*. Furthermore, the proteins related to mitochondrial function and ATP levels in mammary glands were detected. Compared to the control group, the protein levels of DRP1 and FIS1 in mammary glands induced by *K. pneumoniae* were significantly elevated, while the protein levels of MFN1, OPA1, and COX I were markedly reduced (Figure 3H and K–O). ATP levels in the mammary glands infected with *K. pneumoniae* were significantly reduced (Figure 3P). These data indicated that mitochondrial dynamic imbalance, reduction of COX I activity and ATP production have been observed in the bovine mastitis model induced by *K. pneumoniae*.

### *K. pneumoniae* reduced milk fat and protein synthesis in BMECs by inducing mitochondrial dynamic imbalance

**Figure 4.**
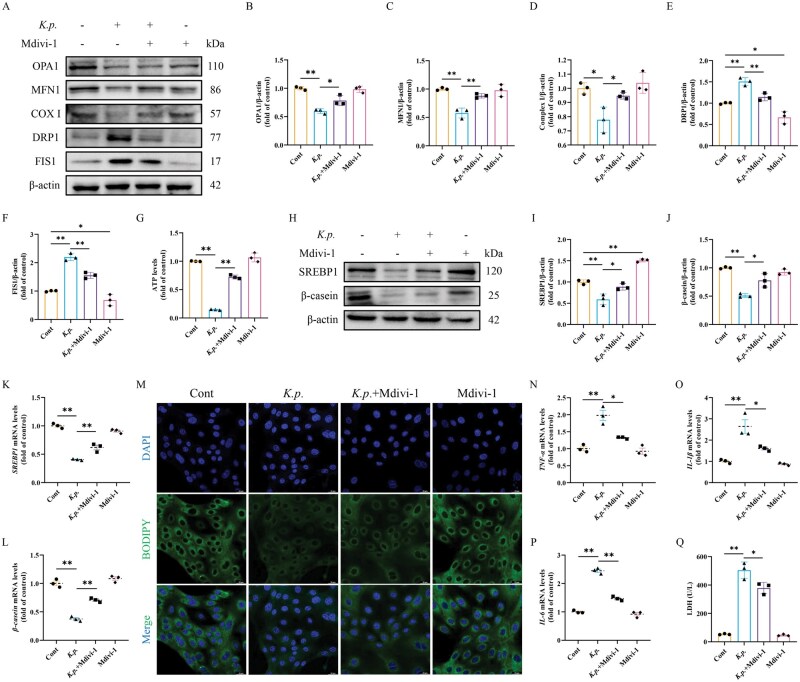
Mdivi-1 recovered *K. pneumoniae*-induced mitochondrial damage and dyssynthesis of milk fat and protein in BMECs. (A) BMECs were treated with *K. pneumoniae* and/or 10 μM Mdivi-1 to analyze the protein levels of OPA1, MFN1, COX I, DRP1, and FIS1. (B–F) Relative protein abundance of OPA1, MFN1, COX I, DRP1, and FIS1 were normalized to β-actin (mean ± SEM, n = 3). (G) Relative ATP levels (mean ± SEM, *n* = 3). (H) BMECs were treated with *K. pneumoniae* and/or 10 μM Mdivi-1 to analyze the protein levels of SREBP1 and β-casein. (I, J) Relative protein abundance of SREBP1 and β-casein were normalized to β-actin (mean ± SEM, *n* = 3). (K, L) BMECs were treated with *K. pneumoniae* and/or 10 μM Mdivi-1 to analyze mRNA levels of *SREBP1* and *β-casein* (mean ± SEM, *n* = 3). (M) Typical images of BODIPY 493/503 staining in BMECs. (N–P) BMECs were treated with *K. pneumoniae* and/or 10 μM Mdivi-1 to analyze mRNA levels of *TNF-α*, *IL-1β*, and *IL-6* (mean ± SEM, *n* = 3). (Q) Detection of the content of LDH (mean ± SEM, *n* = 3). *P < 0.05; **P < 0.01.

To investigate whether *K. pneumoniae* reduced milk fat and protein synthesis by inducing mitochondrial dynamic imbalance, mitochondrial division was inhibited with Mdivi-1. The results showed that the inhibition of mitochondrial division alleviated the decline of MFN1, OPA1, and COX I protein levels significantly and the increase of DRP1 and FIS1 protein levels caused by *K. pneumoniae* infection at MOI of 10 for 6 h (Figure 4A–F). Morever, Mdivi-1 significantly restored the decreased ATP levels caused by *K. pneumoniae* infection with BMECs (Figure 4G). It was suggested that inhibition of mitochondrial division alleviated the mitochondrial dynamic imbalance caused by *K. pneumoniae* infection and enhanced mitochondrial function. Next, we explored whether enhancing mitochondrial function affected milk fat and protein synthesis. The results showed that enhanced mitochondrial function alleviated the decline of milk fat and protein induced by *K. pneumoniae* infection, which was confirmed by the levels of SREBP1 and β-casein genes and proteins (Figure 4H–L). Meanwhile, it has been apparently observed that inhibition of mitochondrial division promoted lipid droplet synthesis (Figure 4M). These results suggested that *K. pneumoniae* infection inhibited milk fat and protein synthesis by inducing mitochondrial dynamic imbalance, and enhancing mitochondrial function promotes milk fat and protein synthesis. Furthermore, we hypothesized that mitochondrial dynamic imbalance induced by *K. pneumoniae* drives inflammatory responses. The mRNA levels of inflammation-related factors showed that the enhancement of mitochondrial function could inhibit the inflammatory response caused by *K. pneumoniae* infection (Figure 4N–P). The results showed that the release of LDH significantly increased after *K. pneumoniae* infection, indicating a decrease in cell viability. The cell viability in the *K. pneumoniae* and Mdivi-1 co-treated group was significantly higher than that in the *K. pneumoniae* group. There was no statistically significant difference in BMECs viability between the Mdivi-1 group and the control group (Figure 4Q). In summary, our findings suggested that *K. pneumoniae* induced dyssynthesis of milk fat and protein as well as inflammatory responses and reduced cell activity by inhibiting mitochondrial function.

### *K. pneumoniae*-induced mammary gland injury was associated with upregulation of FNIP1 expression

**Figure 5.**
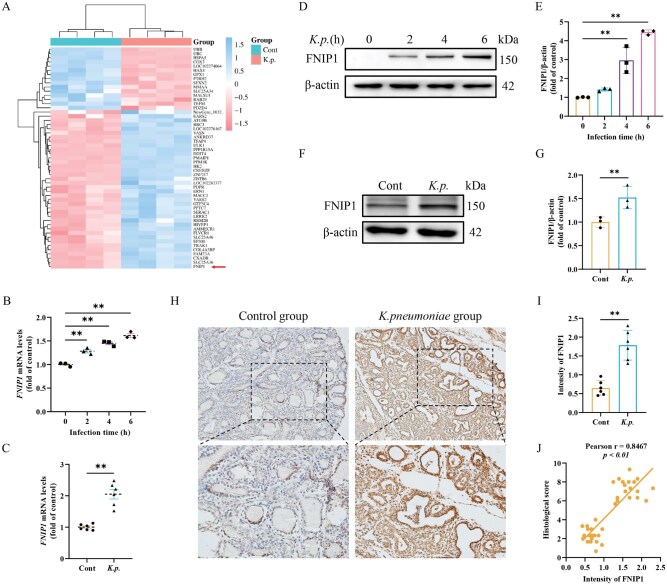
*K. pneumoniae* promoted FNIP1 expression. (A) Heat map of differentially expressed genes of Mitochondrial pathway in the control and *K. pneumoniae* infection groups. (B, C) mRNA levels of *FNIP1* was detected by RT-qPCR method in BMECs (mean ± SEM, *n* = 3) and mammary gland tissues (mean ± SEM, *n* = 6). (D) The protein levels of FNIP1 were detected in BMECs. (E) Relative protein abundance of FNIP1 was normalized to β-actin (mean ± SEM, *n* = 3). (F) The protein levels of FNIP1 were detected in mammary gland tissues. (G) The protein levels of FNIP1 were normalized to β-actin (mean ± SEM, *n* = 3). (H, I) Representative immunohistochemical image and statistical assessment of FNIP1 in the mammary gland tissues sections. (J) Correlations of intensity of FNIP1 with histological scores. *P < 0.05; **P < 0.01.

To further explore the mechanisms underlying mitochondrial dysfunction caused by *K. pneumoniae* infection, we further analyzed the transcriptome of BMECs using RNA sequencing. Compared with the control group, 53 differentially expressed genes related to mitochondrial function were screened in the cells infected with *K. pneumonia*e. Notably, the mitochondrial regulatory factor FNIP1 was upregulated in BMECs infected with *K. pneumoniae* (Figure 5A). Indeed, mRNA and protein levels of FNIP1 increased significantly with the increase of infection time of *K. pneumoniae* in BMECs (Figure 5B, D, and E). Consistent with this, the mRNA and protein levels of FNIP1 were significantly elevated in mammary glands infected with *K. pneumoniae* (Figure 5C, F, and G). Immunohistochemistry results further confirm the increased expression of FNIP1 in mammary glands infected with *K. pneumoniae* (Figure 5H and I). Furthermore, the levels of FNIP1 were positively correlated with histological scores (Figure 5J). Given that, we speculated that FNIP1 may play a role in mitochondrial dynamic imbalance and reduction of COX I activity as well as ΔΨm collapse caused by *K. pneumoniae* infection.

### FNIP1 was involved in *K. pneumoniae*-induced mitochondrial dysfunction

**Figure 6.**
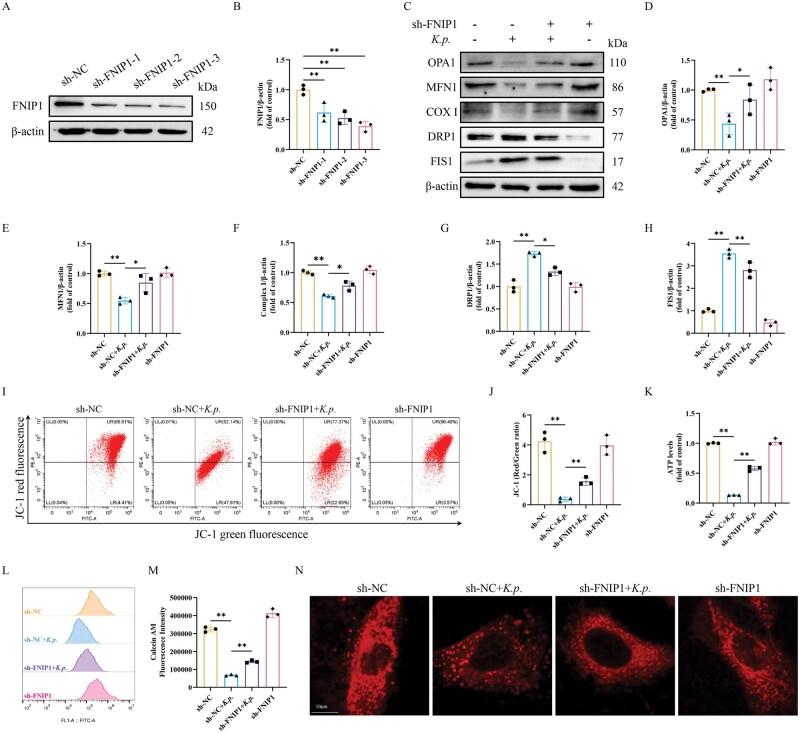
FNIP1 silencing alleviated *K. pneumoniae*-induced mitochondrial dysfunction. (A) BMECs were transfected with control shRNA (sh-NC) and FNIP1 shRNA (sh-FNIP1). The protein levels of FNIP1 was detected in BMECs. (B) Relative protein abundance of FNIP1 was normalized to β-actin (mean ± SEM, *n* = 3). (C) After FNIP1 silence, BMECs were infected with *K. pneumoniae* for 6 h to analyze the protein levels of OPA1, MFN1, COX I, DRP1, and FIS1. (D–H) Relative protein abundance of OPA1, MFN1, COX I, DRP1, and FIS1 were normalized to β-actin (mean ± SEM, *n* = 3). (I) After FNIP1 silence, flow cytometry was used to measure mitochondrial membrane potential level. (J) Changes in mitochondrial membrane potential were determined by the JC-1 red to green fluorescence ratio (mean ± SEM, *n* = 3). (K) Relative ATP levels (mean ± SEM, *n* = 3). (L) The mPTP opening status was evaluated in BMECs by calcein-AM fluorescence. Representative flow cytometry graph and (M) statistical analysis of calcein-AM fluorescence intensity (mean ± SEM, *n* = 3). (N) Mitochondrial morphology of BMECs. The mitochondria were labeled with MitoTracker. *P < 0.05; **P < 0.01.

To further validate the role of FNIP1, FNIP1 shRNA was transfected into BMECs to investigate the effect of FNIP1 on mitochondrial function. It turned out that sh-FNIP1-3 significantly reduced FNIP1 protein levels by 61%, confirming efficient knockdown (Figure 6A and B). As expected, FNIP1 silencing significantly reduced the elevation of DRP1 and FISI caused by *K. pneumoniae* infection at MOI of 10 for 6 h. On the contrary, FNIP1 silencing significantly increased the decrease of OPA1, MFN1, and COX I caused by *K. pneumoniae* infection (Figure 6C–H). Flow cytometry showed that FNIP1 silencing partially rescued the diminished ΔΨm caused by *K. pneumoniae* infection (Figure 6I and J). Meanwhile, FNIP1 silencing significantly recovered the decrease in ATP content caused by *K. pneumoniae* infection (Figure 6K). Additionally, the opening of mPTP caused during *K. pneumoniae* exposure was restored by the silencing of FNIP1(Figure 6L and M). The mitochondrial reticular structure destroyed by *K. pneumoniae* infection was significantly reversed by FNIP1 silencing (Figure 6N). These results suggested that mitochondrial damage caused by *K. pneumoniae* infection is mediated by FNIP1.

### FNIP1 silencing alleviated milk dyssynthesis caused by *K. pneumoniae*

**Figure 7.**
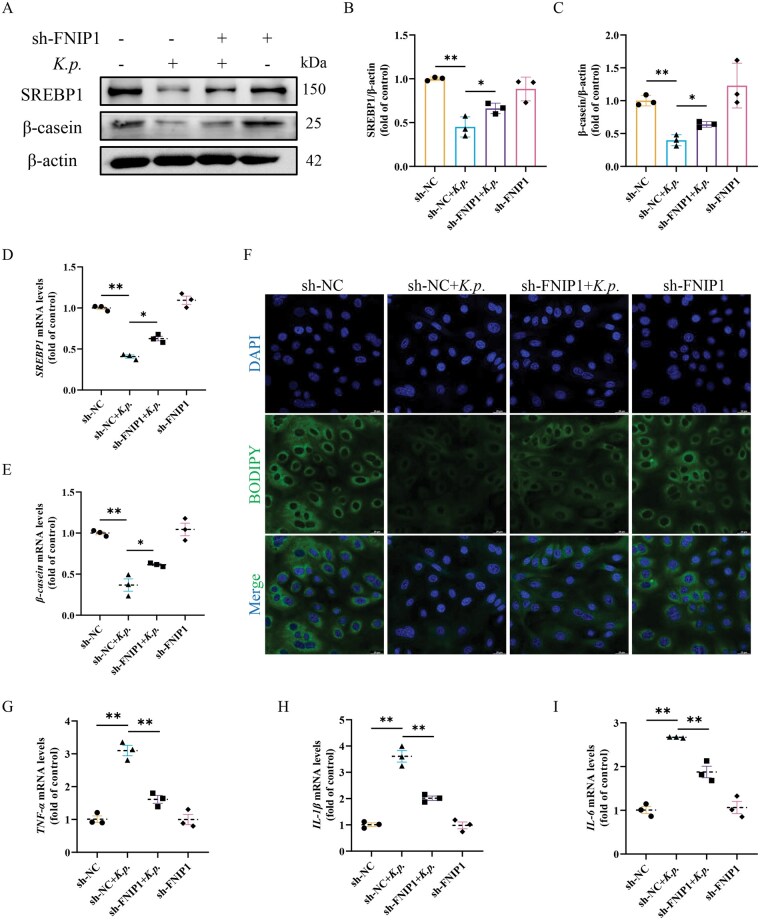
FNIP1 silencing alleviated *K. pneumoniae*-induced milk fat and protein dyssynthesis. (A) After FNIP1 silence, BMECs were infected with *K. pneumoniae* for 6 h to analyze the protein levels of SREBP1 and β-casein. (B, C) Relative protein abundance of SREBP1 and β-casein were normalized to β-actin (mean ± SEM, *n* = 3). (D, E) After FNIP1 silence, mRNA levels of *SREBP1* and *β-casein* were detected by RT-qPCR method in BMECs (mean ± SEM, *n* = 3). (F) Typical images of BODIPY 493/503 staining in BMECs. (G–I) After FNIP1 silence, mRNA levels of *TNF-α*, *IL-1β*, and *IL-6* were detected by RT-qPCR method in BMECs (mean ± SEM, *n* = 3). *P < 0.05; **P < 0.01.

Given the essential role of FNIP1 in mitochondrial function, we investigated whether FNIP1 deficiency affect milk fat and protein synthesis. FNIP1 silencing attenuated the protein reduction of SREBP1 and β-casein induced by *K. pneumoniae* (Figure 7A–C). Similarly, qRT-PCR analysis revealed that FNIP1 silencing reversed *K. pneumoniae*-induced reduction of *SREBP1* and *β-casein* (Figure 7D and E). Furthermore, BODIPY 493/503 staining revealed that FNIP1 silencing recovered the decreased the lipid droplets caused by *K. pneumoniae* infection (Figure 7F). These results suggested that FNIP1 silencing improved milk dyssynthesis caused by *K. pneumoniae*. Notably, FNIP1 silencing reversed the increase of inflammatory factor (*TNF-α*, *IL-1β*, and *IL-6*) mRNA expression induced by *K. pneumoniae* infection, but FNIP1 silenceing alone had no significant effect on the expression of inflammatory factor (Figure 7G–I). These results suggested that FNIP1 silencing attenuated mitochondrial dysfunction-induced milk dyssynthesis through protected mitochondrial damage induced by *K. pneumoniae* infection, thereby suppressing inflammatory factors activation and ultimately mitigating mammary injury.

## Discussion

Previous studies have demonstrated the presence of mitochondrial dysfunction in bovine mammary gland injury caused by *K. pneumoniae* infection (Cheng et al., 2021). Notably, FNIP1 has been identified as a key regulator of mitochondrial function (Malik et al., 2023; Xiao et al., 2024). These findings prompted us to explore whether FNIP1 is associated with *K. pneumoniae*-induced bovine mammary gland injury by regulating mitochondrial function. Based on this consideration, the present study focused on the effects of FNIP1 on *K. pneumoniae*-induced mitochondrial dysfunction, milk fat and protein synthesis, and their triggered inflammatory responses to test the above hypotheses. This study was the first to comprehensively resolve the regulatory role of FNIP1 in *K. pneumoniae*-induced mammary gland damage in dairy cows, providing strong evidence for its use as a potential target for the treatment of mammary gland damage in dairy cows.

*K. pneumoniae* exhibits the capacity to infect diverse cells and tissues, triggering an inflammatory response and leading to cellular damage (Tang et al., 2021; Ma et al., 2022; Zhang et al., 2023). Previous studies have revealed that *K. pneumoniae* is capable of inducing a pro-inflammatory cytokine response in BMECs, as evidenced by the release of cytokines such as TNF-α, IL-1β, and IL-6 (Shi et al., 2021), consistent with our results in a model of BMECs infection. Endotoxin could induce inflammation in BMECs and significantly affect milk production, resulting in decreased milk fat and protein levels per unit volume of milk (Ma et al., 2021; Lyu et al., 2024). Thus, targeted modulation of inflammatory responses in BMECs represents a key therapeutic strategy for mastitis intervention. In a previous study, the impairment of mammary epithelial cells by staphylococcal enterotoxin M was probably related to oxidative stress and mitochondrial damage with decreased mitochondrial membrane potential and intracellular ATP concentration (Zhao et al., 2020). Consistently, BMECs transcriptome analysis revealed that the DEGs mainly occurred in mitochondrial function and lipid metabolism pathways between control and *K. pneumoniae-*infected groups. The imbalance between mitochondrial fission and fusion is closely associated with mitochondrial dysfunction, as evidenced by reduced ΔΨm, diminished respiratory function, and decreased efficiency of oxidative phosphorylation. Notably, COX I, a core component of the electron transport chain, was markedly downregulated in infected BMECs. This downregulation directly impairs ATP synthesis, exacerbating cellular energy deficits and amplifying inflammatory damage. Simultaneously, the expression levels of OPA1 and MFN1, both critical for maintaining mitochondrial network integrity, were significantly downregulated. Conversely, DRP1 and FIS1, key mediators of mitochondrial fission and fragmentation processes, exhibited marked upregulation. These alterations disrupt mitochondrial fusion-fission balance, leading to structural fragmentation and compromised homeostasis. Studies have shown that inflammatory response were closely associated with mitochondrial function, and that damage caused by inflammation can be effectively mitigated by enhancing their function (Bock and Tait, 2020; Marchi et al., 2023; Vringer et al., 2024). Given the crucial role of mitochondria in regulating inflammatory responses, we hypothesize that mitochondrial damage may be act as a driver in *K. pneumoniae*-induced inflammatory response, milk fat and protein dyssynthesis. As expected, *K. pneumoniae*-induced inflammatory response and the concurrent dyssynthesis of milk fat and protein are alleviated by the activation of mitochondrial function, thus enhancing mitochondrial function can be a novel treatment approach for *K. pneumoniae*-induced BMECs damage.

FNIP1 was originally identified as a protein that interacts with folliculin (FLCN) and AMPK (Baba et al., 2006). Using a FLCN-deficient kidney model, it was found that FLCN loss upregulates PGC1α expression, which results in elevated mitochondrial function and oxidative metabolism in renal cancer cells (Hasumi et al., 2012). Emerging evidence establishes FNIP1 as a key molecule in the regulation of mitochondrial function and a scavenging factor for damaged mitochondria (Malik et al., 2023; Xiao et al., 2024). For instance, FNIP1 coordinates mitochondrial biogenesis and quality maintenance (Xiao et al., 2021). Yin et al. showed that FNIP1 deficiency upregulated mitochondrial metabolic genes expression and increased mitochondrial number and volume (Yin et al., 2022). FNIP1 expression was also elevated in BMECs and mammary gland tissues of cows infected with *K. pneumoniae*. Recent studies have identified FNIP1 was involved in the regulation of immune cell developmental processes, muscle fiber type conversion in organisms and influenced the thermogenic remodelling process of adipocytes by regulating mitochondrial function (Reyes et al., 2015; Niehues et al., 2020; Yin et al., 2022). Consistently, inhibition of FNIP1 enhanced mitochondrial function, thereby alleviating *K. pneumoniae*-induced dyssynthesis of milk fat and protein and resultant inflammatory response. The FNIP1 pathway has been shown to influence the cell metabolism regulator AMPK and the mammalian rapamycin target (mTORC1) in multiple cell types (Siggs et al., 2016; Zhou et al., 2017; Ramirez Reyes et al., 2021). For example, FNIP1 regulates mitochondrial energy production mainly through AMPK, which alters the ATP/AMP and ATP/ADP ratios, thereby stimulating mTORC1 signaling and affecting B-cell development (Iwata et al., 2017). Furthermore, inhibition of FNIP1 reactivated AMPK/PGC‐1α signaling and mitochondrial function in myocytes (Liu et al., 2016). FNIP1 (S220) phosphorylation by AMPK participates in the regulation of mitochondrial function in mouse muscle (Xiao et al., 2024). The AMPK signaling pathway serves as a pivotal regulatory axis governing mitochondrial dynamics (Toyama et al., 2016). Specifically, AMPK improves mitochondrial quality control by upregulating OPA1 expression while concurrently negatively regulating Drp1, the primary mediator of mitochondrial fission, through phosphorylation at serine 637 (Li et al., 2015; Zhang et al., 2019; Qiu et al., 2021). Whether the AMPK pathway is related to the mitochondrial damage of BMECs induced by *K. pneumoniae* remains to be elucidated.

The *K. pneumoniae* strain used in this study was isolated from clinical mastitis milk samples, exhibiting HMV phenotype and multidrug resistance. Cheng et al. directly compared HMV strains with non-HMV strains and hypothesized that *K. pneumoniae* strains differ in their ability to damage mitochondria. The results demonstrated that the HMV phenotype of *K. pneumoniae* did not have a key role in causing mitochondrial damage (Cheng et al., 2020, 2021). FNIP1 as a key regulatory hub for mitochondrial damage induced by *K. pneumoniae*, its universality needs to be further verified through comparative studies of strains with different virulence. Especially antibiotic resistance in *K. pneumoniae* is rising, whereas inhibiting FNIP1 may enhance host defense without directly targeting bacteria, potentially reducing resistance pressure. Future research will adopt molecular docking technology to conduct virtual screening of a natural product library, targeting the FNIP1 protein-binding domain to identify high-affinity candidate molecules. Further predict candidate molecular targets, prioritise the exclusion of compounds that strongly bind to key pathways of immune cells, reduce off-target risks, and ensure the specificity of anti-inflammatory effects. Furthermore, liposomal or nanoparticle formulations will be developed to encapsulate FNIP1 siRNA for achieving high intramammary concentration through localized injection, and FNIP1 monoclonal antibody-drug conjugates will be constructed for precise delivery via antibody-mediated specific binding to mammary epithelial cells surface receptors. These strategies achieved localized high-concentration drug administration in mammary tissue while minimizing systemic exposure.

## Conclusions

The underlying mechanism of inhibited mitochondrial function in *K. pneumoniae*-induced inflammatory response is illuminated. The milk fat and protein synthesis inhibited by *K. pneumoniae* depends on FNIP1-mediated mitochondrial dysfunction, which induced an inflammatory response. These conclusions have provided the underlying molecular mechanism of *K. pneumoniae*-induced bovine mammary gland damage, suggesting that FNIP1 is a promising target for control of *K. pneumoniae*-induced bovine mastitis.

## Abbreviations:

BMECs: bovine mammary epithelial cells
COX I: cytochrome c oxidase I
DEGs: differential expressed genes
DRP1: dynamin-related protein 1
FBS: fetal bovine serum
FIS1: fission 1
FLCN: folliculin
FNIP1: folliculin interacting protein 1
H&E: hematoxylin-eosin
HMV: hypermucoviscous
KEGG: Kyoto Encyclopedia of Genes and Genomes
*K. pneumoniae*: *Klebsiella pneumoniae*
MFN1: mitofusin 1
MFN2: mitofusin 2
mPTP: mitochondrial permeability transition pore
OPA1: optic atrophy 1
SREBP1: sterol regulatory element binding protein 1
TEM: transmission electron microscopy
ΔΨm: mitochondrial membrane potential
